# Changes in the Use of Intensive and Supportive Care with Heart Failure in the Last Month of Life Between 2001 and 2013

**DOI:** 10.1093/geroni/igab046.2322

**Published:** 2021-12-17

**Authors:** Piling Chou, Peichao Lin

**Affiliations:** 1 Kaohsiung Medical University, Kaohsiung, Taiwan, Kaohsiung, Taiwan (Republic of China); 2 Kaohsiung Medical University, Kaohsiung, Kaohsiung, Taiwan (Republic of China)

## Abstract

Background: Heart failure (HF) is a global epidemic affecting the elder globally. It is uncertain what care patients with heart failure receive at their end of life and what care trends are in the last month of life. OBJECTIVES: This study's objective was to investigate the changes in the use of intensive and supportive procedures for Taiwanese patients with heart failure in their last month of life during 2001-2013. METHODS: Analysis of claims data of 25,375 patients with heart failure obtained from the National Health Insurance Research Database was performed to investigate the changes in the use of intensive and supportive procedures for Taiwanese patients with heart failure in their last month of life during 2001-2013. RESULTS: Over the whole study period, 53.3% of patients with heart failure were admitted to intensive care units in their last month of life. The percentages of patients receiving mechanical ventilation (54.3%-41.5%), cardiopulmonary resuscitation (41.5%-16.7%), decreased over time. The percentages of patients receiving artificial hydration and nutrition (52.5.9%-56.8%) and extracorporeal membrane oxygenation(ECMO) (0.52%-1.78%) increased over time. Patients under 75 years old were more likely to be admitted to intensive care units. CONCLUSION: Over time, supportive procedures increased, and intensive procedures decreased in patients with heart failure in the last month of life. This study highlights a need for research, guidelines, and training in how to provide palliative care for end-stage patients with heart failure.

